# A rare case of abdominal pain and hematochezia: case report and literature review

**DOI:** 10.3389/fmed.2025.1637720

**Published:** 2025-10-21

**Authors:** Wenjuan Guo, Min Ling, Yanfen Shi, Minggang Zhang, Xiaoyun Cheng, Jianan Chen, Xiaodi Wang, Shiyu Du

**Affiliations:** ^1^Department of Gastroenterology, China-Japan Friendship Hospital, Beijing, China; ^2^The First Clinical School of Medicine, Zhengzhou University, Zhengzhou, Henan, China; ^3^Department of Pathology, China-Japan Friendship Hospital, Beijing, China; ^4^Department of Clinical Sciences, H. Lee Moffitt Cancer Center & Research Institute, Tampa, FL, United States

**Keywords:** idiopathic myointimal hyperplasia, mesenteric veins, abdominal pain, hematochezia, colonic ischemia, case report, misdiagnosis

## Abstract

Idiopathic myointimal hyperplasia of the mesenteric veins (IMHMV) is a rare, non-thrombotic, non-inflammatory cause of chronic colonic ischemia, often misdiagnosed as inflammatory bowel disease (IBD) or ischemic colitis due to overlapping clinical features. We present the case of a 65-year-old man with progressive abdominal pain and hematochezia, initially treated as ischemic colitis without improvement. Surgical resection was eventually performed, and definitive diagnosis of IMHMV was established through histopathological evaluation, which revealed characteristic intimal smooth muscle proliferation in mesenteric veins. This report reviews the diagnostic challenges, histologic features, and clinical relevance of IMHMV, emphasizing the importance of early recognition and consideration of this entity in refractory colonic ischemia. Routine use of vascular imaging may help distinguish IMHMV from occult mesenteric arteriovenous malformations.

## Introduction

Idiopathic myointimal hyperplasia of the mesenteric veins (IMHMV) is a rare and under-recognized vascular disorder characterized by non-thrombotic, non-inflammatory occlusion of small-to-medium-sized mesenteric veins due to smooth muscle proliferation within the intima (1). First described by Genta and Haggitt (2), IMHMV typically presents with progressive abdominal pain, hematochezia, diarrhea, and signs of chronic colonic ischemia, predominantly affecting middle-aged to elderly men. Despite its benign histological nature, IMHMV is often misdiagnosed as inflammatory bowel disease or ischemic colitis due to overlapping clinical and endoscopic features (3).

Accurate preoperative diagnosis is challenging, as conventional endoscopic biopsies and imaging studies often yield nonspecific findings. Definitive diagnosis usually requires surgical resection and histopathological confirmation (4). Early recognition is critical, as conservative therapy is ineffective and delayed diagnosis may lead to severe complications such as bowel perforation or massive hemorrhage (5).

This report describes a case of IMHMV in a 65-year-old male with a history of left hemicolectomy, who presented with persistent hematochezia and progressive abdominal pain. We discuss the diagnostic challenges, histopathological features, and differential considerations, and we highlight emerging evidence suggesting a potential role of occult mesenteric arteriovenous fistulas in the pathogenesis of this condition (Table 1).

**Table 1:** Comparison of IMHMV with its principal mimickers.

Feature	IMHMV	IBD	Ischemic colitis	MIVOD
Typical age	50–70 y	15–40 y	> 60 y	40–60 y
Common site	Rectosigmoid	Rectum; Colon	Splenic flexure/sigmoid	Rectosigmoid
Pathology	SM proliferation in veins	Mucosal; Transmural inflammation	Mucosal hemorrhage, necrosis	Lymphocytic venulitis, thrombosis
Steroid response	None	Good	Minimal	Poor
Endoscopic	Circumferential ulcer	Continuous; Skip lesions	Segmental ulcers	Patchy, IBD-like
Imaging clue	Tortuous engorged mesenteric veins	Comb sign, mural enhancement	Thumb-printing, low arterial flow	Venous wall thickening
Treatment	Embolization preferred	Immunosuppressants, biologics	Supportive; surgery if complicated	Surgery (curative)

MIVOD, mesenteric inflammatory veno-occlusive disease; SM, smooth muscle.

## Case presentation

A 65-year-old man with a history of left hemicolectomy for descending colon cancer, hypertension, and type 2 diabetes mellitus presented with progressively worsening abdominal symptoms. He was diagnosed with descending colon adenocarcinoma and underwent left hemicolectomy 24 months prior to the current admission. Histopathologic evaluation of the surgical specimen revealed a moderately differentiated adenocarcinoma, staged as pT3N0M0 (Stage IIA), with negative margins. Two months prior to admission, he developed sudden onset of lower abdominal distension and pain (VAS score 5–6), accompanied by the passage of yellow watery stools. His diarrhea worsened over the following weeks, reaching 5–6 episodes daily and was associated with tenesmus and postprandial abdominal pain. Ten days before referral, his symptoms acutely deteriorated, with diarrhea increasing to 20 episodes per day and the appearance of dark red blood in the stool.

**Figure 1:**
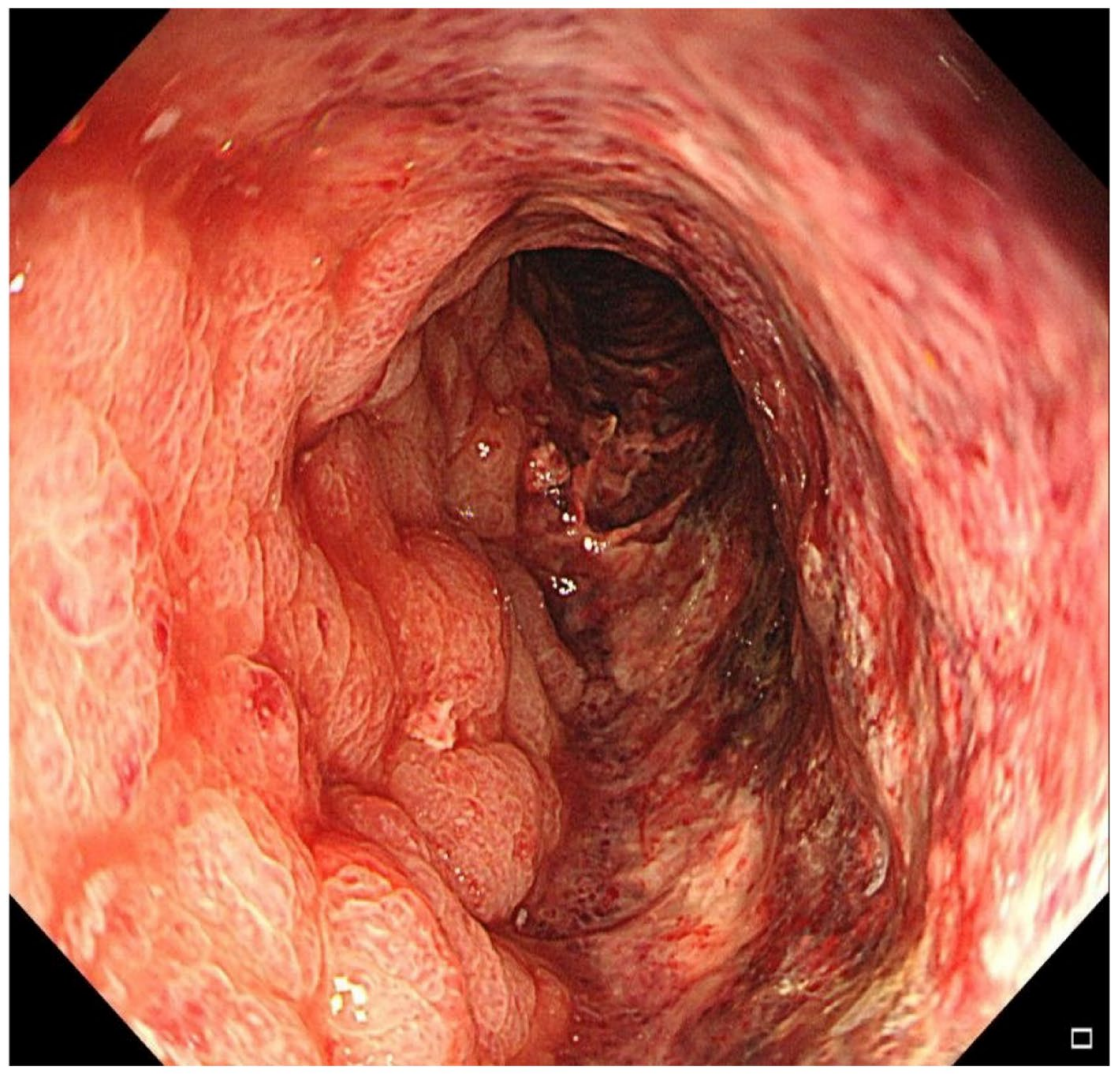
Colonoscopy demonstrated diffusely edematous and violaceous mucosa extending from 15 cm to 30 cm above the anal verge, with friability and granular appearance.

Initial colonoscopy performed at a local hospital revealed circumferential mucosal congestion, edema, and erosion in the sigmoid colon (15–30 cm from the anal verge), a visible anastomosis site was noted at 35 cm from the anal verge. Histopathology showed chronic inflammation with vascular dilation. A preliminary diagnosis of ischemic colitis was made. He was treated with bowel rest, intravenous fluids, mesalazine, and antibiotics, but his condition worsened—abdominal pain intensified (VAS 7–9), and bloody diarrhea increased to 30–40 episodes per day. He was subsequently referred to our hospital for further evaluation. On admission, physical examination revealed abdominal distension, lower abdominal tenderness, and marked rebound pain. Laboratory investigations showed leukocytosis [WBC 10.87 × 10⁹/L (3.5–9.5)], elevated D-dimer [1.8 mg/L FEU (reference <0.5 mg/L FEU)], hyponatremia [Na^+^ 131 mmol/L (135–145)], and hypokalemia [K^+^ 3.2 mmol/L (3.5–5.1)]. Repeat colonoscopy demonstrated diffusely swollen, purple mucosa extending from 15 to 30 cm above the anal verge (Figure 1). Biopsy again revealed chronic inflammation with mucosal necrosis. Serological tests for autoimmune diseases (ANA, ANCA, anticardiolipin antibodies) and infectious pathogens (CMV-IgM, *Clostridium difficile*) were all negative.

**Figure 2:**
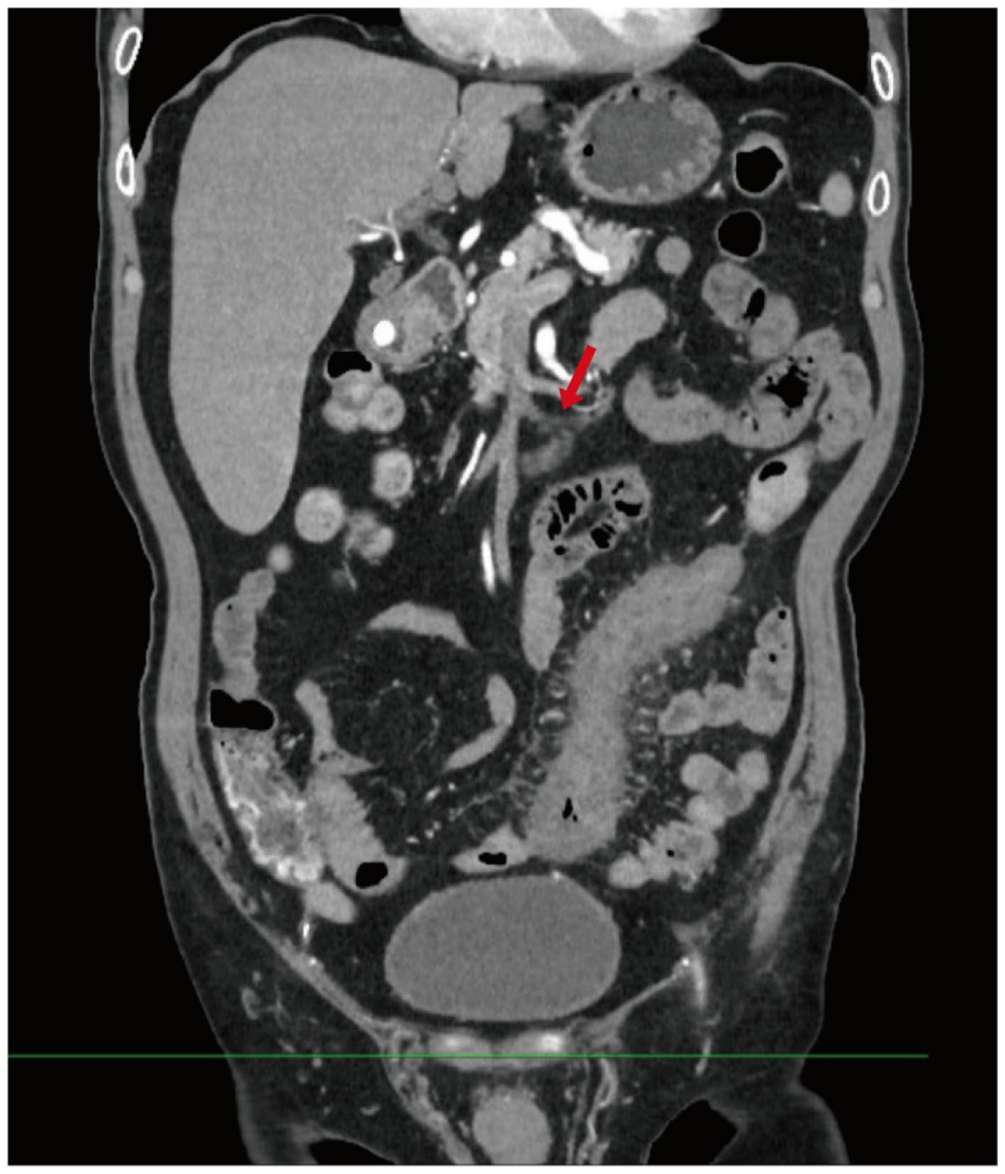
Contrast-enhanced abdominal CT. This image shows diffuse bowel wall thickening and mucosal enhancement involving the descending and sigmoid colon, with dilation of several adjacent bowel loops. Multiple engorged and tortuous mesenteric vessels are visualized, along with mild stranding of the surrounding mesenteric fat (Red arrow indicates tortuous mesenteric veins).

**Figure 3:**
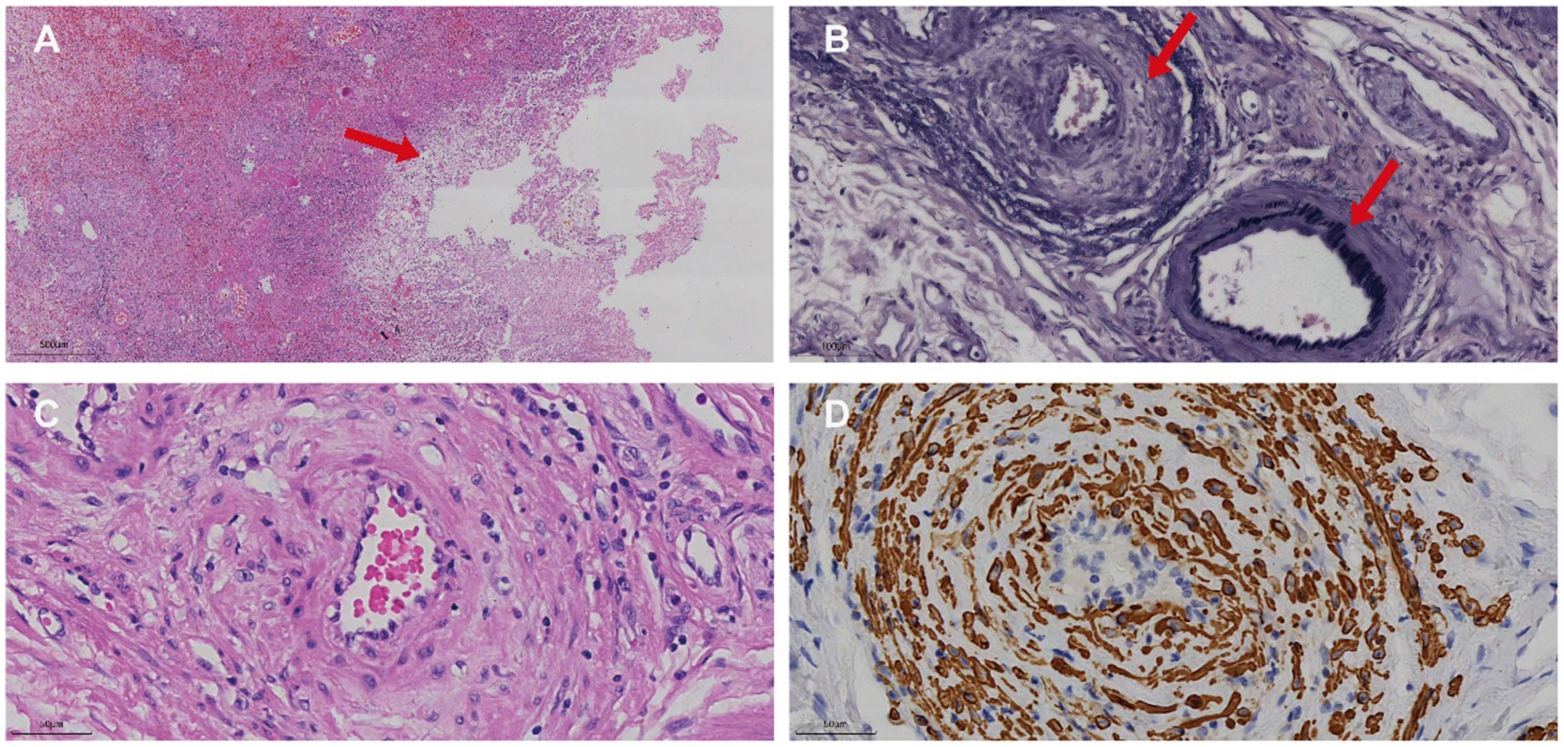
Histopathological and immunohistochemical features of IMHMV. **(A)** Low-power H&E image of the affected colon showing mucosal necrosis (arrow) associated with ischemic changes. **(B)** High-power elastin stain demonstrating a normal mesenteric artery with an intact black internal elastic lamina (lower arrow) and an adjacent vein lacking an elastic lamina but exhibiting marked intimal thickening (upper arrow). **(C)** High-power H&E section showing concentric intimal hyperplasia within the venous wall, leading to luminal narrowing. **(D)** Corresponding high-power SMA immunohistochemistry confirming proliferation of smooth muscle cells within the intima of the affected vein.

**Figure 4:**
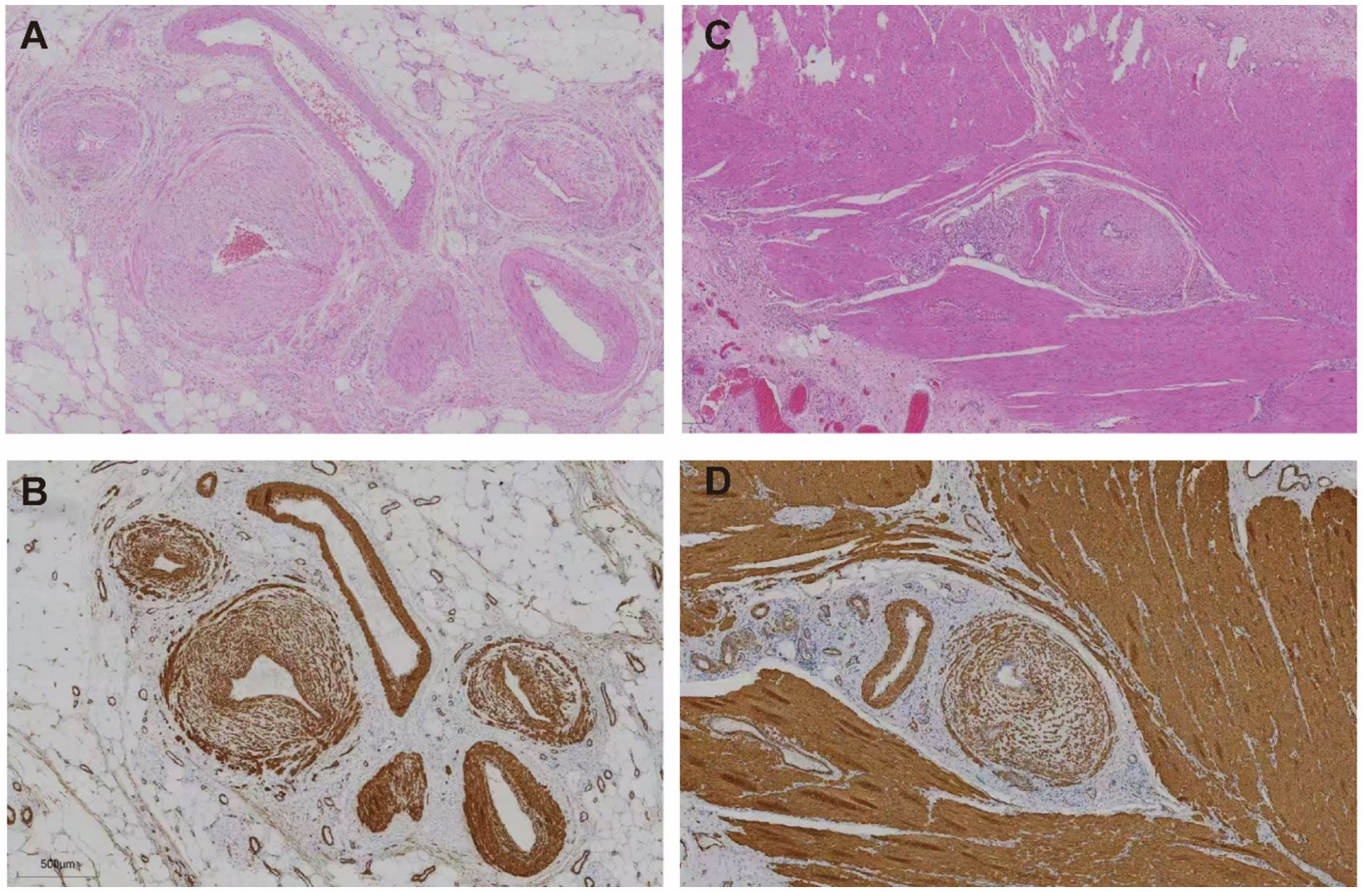
Histological features of affected colonic veins. **(A,B)** Subserosal layer: **(A)** Hematoxylin and eosin (H&E) staining reveals concentric intimal hyperplasia of small veins with luminal narrowing. **(B)** Smooth muscle actin (SMA) immunostaining highlights prominent smooth muscle proliferation within the venous intima. **(C,D)** Muscularis propria: **(C)** H&E staining shows thickened vascular walls embedded in muscle fibers. **(D)** SMA staining confirms diffuse smooth muscle proliferation within the intima of intramuscular veins.

Contrast-enhanced abdominal CT revealed significant bowel wall edema distal to the previous anastomosis site, suggestive of ischemia (Figure 2). Given the clinical course and inconclusive findings from conservative management, the surgical team proceeded with exploratory laparotomy. The patient underwent a redo left-sided colectomy with resection of the prior anastomosis and adhesiolysis, followed by reconstruction with a new colorectal anastomosis. Intraoperative findings included dark purple discoloration and mesenteric ischemia involving the bowel from 10 cm above the anal verge to the mid-sigmoid colon, without evidence of arterial occlusion or torsion. Histopathological examination revealed extensive acute and chronic inflammation, edema, and ulceration of the bowel wall. Notably, marked smooth muscle proliferation within the intima of mesenteric veins, leading to luminal narrowing or occlusion, was observed from mucosa to serosa. In contrast, the mesenteric arteries in the submucosa and subserosa were structurally preserved, with intact elastic laminae and no evidence of inflammation, thrombosis, or intimal hyperplasia. Representative histopathological images are shown in Figure 3, including a low-power mucosal view with ischemic ulceration (panel A), high-power elastin stain differentiating uninvolved arteries from affected veins (panel B), high-power H&E showing concentric venous intimal hyperplasia (panel C), and corresponding smooth muscle actin (SMA) immunohistochemistry confirming proliferation of intimal smooth muscle cells (panel D). Elastic fiber staining and immunohistochemistry for SMA confirmed the diagnosis of idiopathic myointimal hyperplasia of mesenteric veins (Figure 4). No evidence of malignancy was found in the resected bowel or in 22 reactive mesenteric lymph nodes. Postoperatively, the patient’s symptoms improved rapidly. Bloody diarrhea resolved by postoperative day 3, liquid diet was resumed on day 7, and serum electrolytes and D-dimer levels normalized by day 14. He was subsequently discharged in stable condition.

## Discussion

IMHMV is a rare, non-thrombotic, non-inflammatory cause of venous ischemia in the bowel, most commonly affecting the left colon—especially the rectosigmoid segment (6). The disease is characterized histologically by proliferation of smooth muscle within the intima of small-to-medium-sized mesenteric veins, leading to luminal narrowing and resultant ischemic injury. While its precise pathogenesis remains unclear, several theories have been proposed, including chronic mechanical stress, arteriovenous flow disturbances, and repetitive trauma to the mesentery (7).

Clinically, IMHMV predominantly affects middle-aged or elderly men and typically presents with progressive abdominal pain, hematochezia, diarrhea, and tenesmus. Due to its overlap with inflammatory bowel disease (IBD), IMHMV is often misdiagnosed and initially treated with corticosteroids or other IBD-directed therapies, which are ineffective (8). A more detailed comparison with inflammatory bowel diseases is warranted, given the frequent misdiagnosis of IMHMV. Clinically, IMHMV patients usually present with continuous left-sided colonic involvement, whereas ulcerative colitis typically begins in the rectum and Crohn’s disease often shows discontinuous “skip lesions.” Endoscopically, IMHMV demonstrates circumferential edema and purple discoloration without the aphthous ulcers or cobblestoning characteristic of Crohn’s disease. Histologically, IMHMV lacks the crypt abscesses, basal plasmacytosis, and transmural lymphoid aggregates commonly observed in IBD. Furthermore, corticosteroids and other IBD-targeted therapies are ineffective in IMHMV, underscoring the importance of recognizing these distinctions for timely surgical intervention. In our case, the patient presented with worsening hematochezia and non-resolving diarrhea, was initially diagnosed with ischemic colitis, and ultimately required surgery due to failed conservative management.

Endoscopic features often include circumferential or longitudinal ulcerations, mucosal fragility, and a cobblestone appearance. Notably, rectal sparing and continuous lesion distribution (without “skip areas”) are distinguishing features from Crohn’s disease. Although endoscopic biopsies are usually insufficient for definitive diagnosis, certain features—such as thickened, arterialized capillaries with sub-endothelial fibrin deposition—may raise suspicion for IMHMV (9). Radiological findings may support this diagnosis as well; segmental bowel wall thickening, mucosal edema, and mesenteric venous abnormalities have been reported in some cases (10).

The diagnosis is typically confirmed postoperatively via histopathological examination of the resected specimen, which demonstrates characteristic intimal smooth muscle proliferation without inflammation (11). IMHMV must be differentiated from mesenteric inflammatory veno-occlusive disease (MIVOD), which is defined by lymphocytic venulitis. Some investigators have proposed that MIVOD and IMHMV may represent different points along a spectrum of the same disease (12, 13). In our case, the absence of inflammatory infiltrates and confirmation of intimal hyperplasia by elastic staining and SMA immunohistochemistry supported a diagnosis of IMHMV.

Surgical resection remains the only curative treatment, as no effective medical therapy currently exists. Most patients show rapid resolution of symptoms postoperatively, as was observed in our case. Therefore, prompt surgical consultation is essential in cases of treatment-refractory colonic ischemia, particularly in middle-aged or older men with left-sided colonic involvement.

However, the nature of IMHMV as a distinct clinicopathological entity has recently been challenged. Rozner et al. (1) conducted a comprehensive review highlighting the importance of recognizing IMHMV in atypical ischemic colitis presentations, particularly in younger males with rectosigmoid involvement. Nevertheless, some authors contend that many of the reported IMHMV cases may actually represent unrecognized mesenteric arteriovenous fistulas (AVFs) or malformations. These AVFs are believed to induce venous hypertension and arteriovenous shunting, resulting in secondary ischemia and reactive myointimal hyperplasia. Sterpetti et al. (14) have proposed that such hyperdynamic blood flow environments can create zones of shear stress variation, triggering inflammation and vascular remodeling. In this context, it is important to differentiate IMHMV from mesenteric arteriovenous dysplasia or vasculopathy. Arteriovenous dysplasia typically involves direct arteriovenous communications or vascular malformations that create a high-flow state, resulting in venous hypertension, turbulent shear stress, and compensatory vascular remodeling. In contrast, IMHMV is defined by isolated smooth muscle hyperplasia of the venous intima without evidence of arteriovenous shunting or arterial involvement. While both entities may present with similar clinical features and ischemic changes, AV dysplasia usually demonstrates early venous filling on angiography and may be amenable to endovascular embolization. Histologically, arteries in IMHMV appear structurally intact, whereas arteriovenous dysplasia may show distorted or hyperplastic arterial–venous junctions. Thus, accurate distinction between the two is essential, as their underlying pathophysiology and treatment strategies differ significantly.

The identification of AVFs can be extremely challenging. Even advanced cross-sectional imaging (CT and MR angiography) may fail to detect them. In the review by Rozner et al. (1) only one-third of patients underwent cross-sectional angiography, and just 5% (seven patients) underwent dynamic digital subtraction angiography (DSA). It is therefore plausible that a substantial proportion of cases labeled as IMHMV may in fact be misdiagnosed AVF-related ischemia.

A notable case by Hendy et al. reported a young male with severe rectosigmoid ischemic colitis who had normal CT and MR angiography but was ultimately found to have a subtle mesenteric AVF on DSA (15). The patient was treated successfully with embolization, avoiding the need for surgery. This case emphasizes the importance of thorough vascular imaging in suspected IMHMV cases.

Accordingly, we propose that the term “idiopathic” IMHMV should be reserved for patients in whom mesenteric AVMs have been rigorously excluded using DSA. Future diagnostic algorithms, including those proposed by Rozner et al. (1) would benefit from revision to incorporate routine use of DSA in suspected cases, and to consider curative embolization as a definitive therapeutic option prior to surgery.

In conclusion, while IMHMV remains a rare but important differential diagnosis in chronic colonic ischemia, evolving evidence suggests that many cases may have a vascular etiology that is potentially amenable to endovascular intervention. Multidisciplinary approaches and careful imaging are essential to ensuring accurate diagnosis and optimal management. Based on our experience and the available literature, we recommend the following diagnostic and management approach for similar future cases. First, a thorough diagnostic work-up should be performed, including contrast-enhanced CT, followed by digital subtraction angiography when IMHMV is suspected, as endoscopic biopsies are usually nonspecific. Colonoscopy may demonstrate continuous mucosal changes without skip lesions, but histology from limited biopsies is often insufficient, necessitating surgical pathology for definitive diagnosis. Second, conservative medical therapies such as corticosteroids or mesalamine are ineffective; thus, prompt surgical consultation is advised once refractory ischemic colitis or suspected IMHMV is encountered. In selected cases with identifiable arteriovenous fistulas, endovascular embolization may be considered as a less invasive option. Third, perioperative management should focus on correcting electrolyte disturbances, optimizing nutritional status, and closely monitoring for complications such as perforation or massive bleeding. Finally, follow-up should include regular clinical assessments and imaging as appropriate to monitor for recurrence or vascular abnormalities, as well as evaluation of postoperative bowel function and quality of life.

## Data Availability

The original contributions presented in the study are included in the article/supplementary material, further inquiries can be directed to the corresponding authors.
